# The effects of surface wettability on the fog and dew moisture harvesting performance on tubular surfaces

**DOI:** 10.1038/srep24276

**Published:** 2016-04-11

**Authors:** Donghyun Seo, Junghun Lee, Choongyeop Lee, Youngsuk Nam

**Affiliations:** 1 Department of Mechanical Engineering, Kyung Hee University, Yongin 446-701, Korea

## Abstract

The efficient water harvesting from air-laden moisture has been a subject of great interest to address world-wide water shortage issues. Recently, it has been shown that tailoring surface wettability can enhance the moisture harvesting performance. However, depending on the harvesting condition, a different conclusion has often been reported and it remains unclear what type of surface wettability would be desirable for the efficient water harvesting under the given condition. Here we compare the water harvesting performance of the surfaces with various wettability under two different harvesting conditions–dewing and fogging, and show that the different harvesting efficiency of each surface under these two conditions can be understood by considering the relative importance of the water capturing and removal efficiency of the surface. At fogging, the moisture harvesting performance is determined by the water removal efficiency of the surface with the oil-infused surfaces exhibiting the best performance. Meanwhile, at dewing, both the water capturing and removal efficiency are crucial to the harvesting performance. And well-wetting surfaces with a lower barrier to nucleation of condensates exhibit a better harvesting performance due to the increasing importance of the water capture efficiency over the water removal efficiency at dewing.

As the demand for the clean water is increasing in the world, tapping on the hitherto un-used water source –moisture in air – has been proposed as a simple and low cost approach to address a water shortage problem^1^. Broadly, air-laden moisture can be harvested in two different ways. One collects air-suspended tiny droplets under fog directly, e.g. by intercepting tiny droplets using the vertically placed nets when they pass through the nets^2^,^3^,^4^. Another captures the air-laden moisture via vapor condensation on the surface^5^,^6^,^7^. In the present study, the first and second mode of the moisture harvesting is termed as “fog harvesting” and “dew harvesting”, respectively.

Previous studies have demonstrated that the water collection efficiency is strongly correlated with the surface wettability as well as structural features on the surface in both fog and dew harvesting^8^,^9^. In particular, motivated by certain species found in an arid area, various biomimetic artificial surfaces have been developed by incorporating the similar wettability and roughness features as their natural counterparts^8^. For example, the surfaces with the mixed wettability patterns of hydrophilicity and hydrophobicity, mimicking the desert beetle’s back^10^,^11^, have exhibited a better fog harvesting performance compared with the surface with a homogeneous wettability^12^,^13^,^14^,^15^,^16^. Also, bioinspired fibers with spindle knots or diameter variation, as observed in spider webs^17^,^18^ or cactus spine^19^,^20^, have been shown to be an excellent fog collector due to the directional transport of the collected water droplet driven by Laplace pressure difference. Recently, the fog harvesting potentials of oil-infused micro/nanostructured surfaces have been explored for the high droplet mobility on these surfaces^21^.

Although those studies have demonstrated that the surface modification could be an effective way to enhance the performance of the moisture harvesting surface, the surface with similar wettability often exhibits the opposite performance in fog and dew harvesting^12^,^15^,^22^. For example, while non-wetting surfaces generally showed the better fog harvesting performances, they performed poorly in dew harvesting^15^,^22^. Furthermore, in actual experiments, it has been observed that the effectiveness of the surface modification in the moisture harvesting is often influenced by the chemical/physical edges present on the surface as well as the tilting angle of the surface^15^,^22^. For example, the previous study showed that the heterogeneous wettability of hydrophilicity and hydrophobicity can deteriorate the harvesting performance when water droplets are pinned at boundaries between hydrophobic and hydrophilic regions^22^. Also, in the same study, it has been demonstrated that the performance of the hydrophilic surfaces is strongly influenced by the edge condition of the collection surface and by relaxing the pinning of draining water at the edge of the hydrophilic surface using the chemical patterning, they could increase the amount of the harvested water significantly^22^. In another study, it has been shown that the superhydrophilic surface exhibits the better dew harvesting performance over superhydrophobic surface and mixed superhydrophobic/superhydrophilic surface only when the tilting angle is sufficiently high (i.e. >60°)^15^.

These results suggest that the moisture harvesting performance of the modified surface is strongly influenced by the harvesting condition (fogging or dewing) as well as other variations introduced along with the wettability change such as a pinning strength. However, due to the difference of the surface preparation method as well as the environmental condition including a super-saturation level, it is difficult to reach the definite conclusion about the effect of the surface wettability on the moisture harvesting performance from the existing literature. Hence, in the present study, to clarify the effect of the surface wettability on the moisture harvesting, we quantify the influence of the surface wettability on water harvesting performance under the two different harvesting conditions of fogging and dewing, while excluding the influence of the meniscus pining associated with the physical edge by employing a tubular surface, which is also relevant to many industrial applications such as the multi stage flash (MSF) desalination^23^,^24^,^25^. Also, from the measurement of the mass and frequency of the collected water from the surface, we propose the rational explanation for the obtained results for fog harvesting and dew harvesting.

To modify the surface property of tubular surfaces, we employ the surface modification technique based on chemical oxidation of Cu following our previous studies^26^,^27^. Under proper conditions, CuO nanostructures with blade-like morphology can be grown over Cu surfaces, which exhibit superhydrophobic properties after a coating with low surface energy material of Silane. Also, with an infusion of a low surface tension oil into nanostructures, water repellent oil-infused surfaces with low contact angle hysteresis can be created. Due to the easier removal of water droplets from superhydrophobic and oil-infused surfaces, both surfaces are believed to hold a great potential for thermal applications involving vapor-water phase change^28^,^29^,^30^,^31^,^32^. Here we use the same surface modification technique and study how such a surface modification leads to the different water collecting results in fog harvesting and dew harvesting. Particularly, in agreement with our previous study with a flat plate^9^ and other studies with directional droplet transport, we show that an efficient water removal from the collecting site (or a faster surface regeneration) is a key factor for a good fog harvesting performance. In comparison, in dew harvesting, we demonstrate that the nucleation barrier to condensation should be additionally considered, as the water capture efficiency becomes more important for the harvesting performance at dewing.

## Results and Discussions

### Experimental setup and sample fabrication

**Figure 1:**
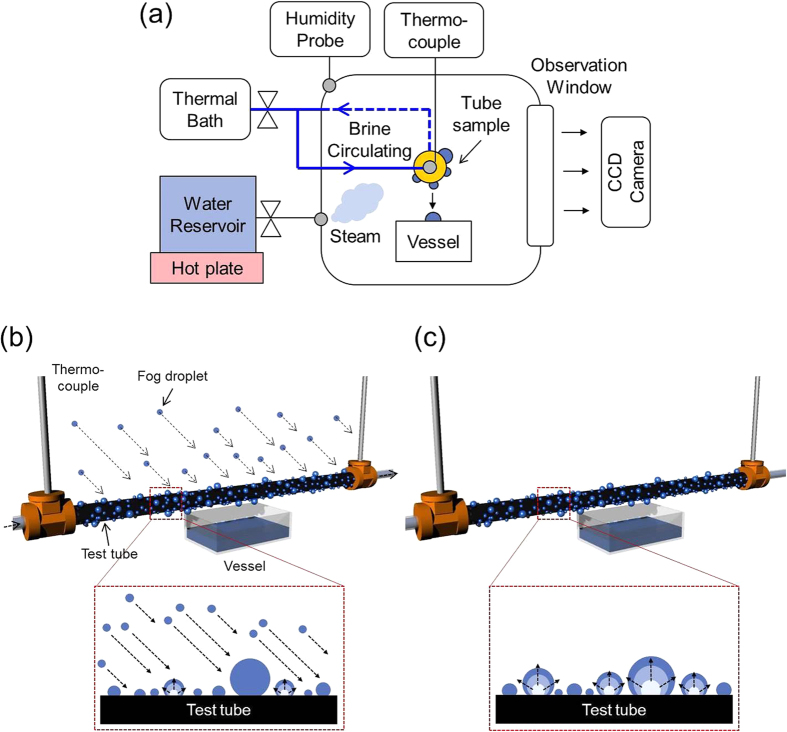
(**a**) Schematics of the water harvesting setup for fog harvesting and detailed schematics of (**b**) fog harvesting process and (**c**) dew harvesting process. In fog harvesting, hot vapor generated from the boiling water is supplied directly to the test sample. Hence, water droplets form and grow mainly by collision with tiny saturated droplets in steam and condensation. (**c**) In dew harvesting, there is no supplied vapor stream into the surface and the water is captured only via condensation.

**Figure 2:**
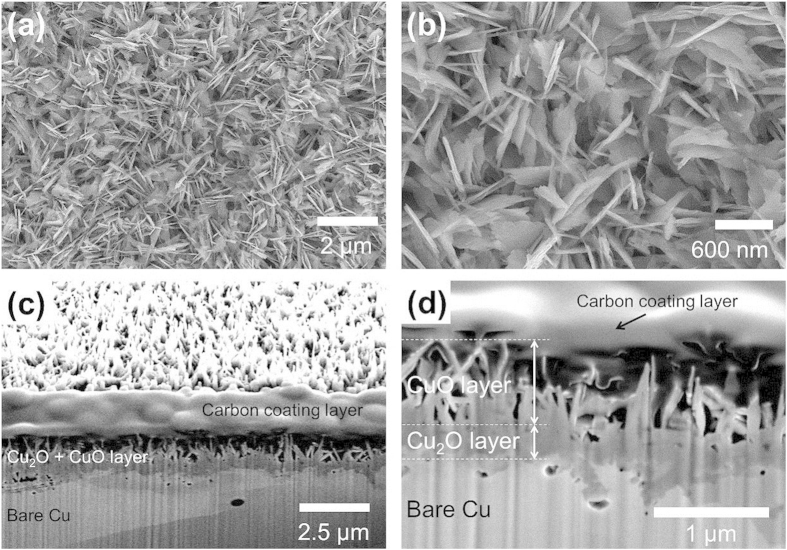
(**a,b**) SEM images of top view of a nanostructured CuO surface. (**c,d**) SEM images of side view of a FIB milled nanostructured CuO surface. The thicknesses of CuO and Cu_2_O layers are about 800 nm and 300 nm, respectively.

Figure 1a shows a schematic of the water harvesting setup for fog harvesting, while Fig. 1b,c show the detailed schematics of the fog and dew harvesting process on a tubular surface. In fog harvesting, a hot vapor generated from the boiling water is directly supplied to the test sample and the surface captures the water via a collision with saturated droplets in air as well as via condensation. In dew harvesting, there is no supplied vapor stream into the surface and the water is captured only via condensation. As the test surfaces, we used commercially available Cu tubes for the aforementioned reasons. Various wetting characteristics were successfully embedded onto Cu tubes through a chemical oxidation and a vapor deposition processes (denoted as Bare: w/o both chemical oxidation and vapor deposition, HPo: w/vapor deposition only, SHPi: w/chemical oxidation only, SHPo: w/both chemical oxidation and vapor deposition). Figure 2 shows SEM images of such fabricated nanostructures on Cu surface. Additionally, oil-infused surfaces, which have recently attracted much attention for their robust liquid repellency, were prepared by infusing the Krytox 5cst oil or Silicone oils with different viscosities (5, 100, 1000cst) to SHPo surface. In the present study, each oil infused surface was denoted with their type and viscosity such as K.Oil 5cst (5cst krytox oil infused), S.Oil 5cst (5cst silicone oil infused), S.Oil 100cst (100cst silicone oil infused), and S.Oil 1000cst (1000cst silicone oil infused). Further experimental details can be found in the Methods and Supplementary Fig. S1.

### Characterization of surface wettability

**Figure 3:**
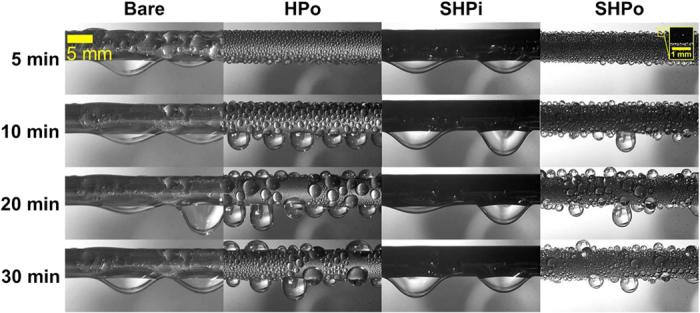
Time-lapse images of the fog harvesting on Bare, HPo, SHPi, and SHPo surfaces. On the Bare and SHPi, the outer surface of tubes is covered with a liquid film, while dropwise water capturing process occurs on HPo and SHPo surfaces. Particularly, the SHPo surface has a temporally different droplet behavior; in the beginning, it is seen that numerous tiny droplets (<50 μm) jump away from the surface, while a droplet jumping event disappears soon due to the flooding of nanostructures under the high super-saturation condition (*S* = 7.5−7.6).

**Figure 4:**
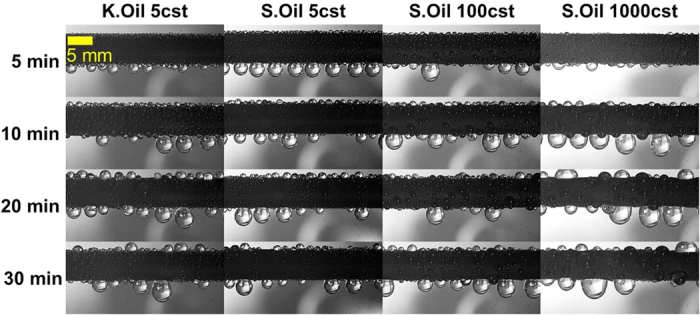
Time-lapse images of the fog harvesting on K.Oil 5cst, S.Oil 5cst, S.Oil 100cst, S.Oil 1000cst, and S.Oil 1000cst surfaces. Contrary to the SHPo surface, the droplet shedding dynamics is not influenced by the high super-saturation condition due to the presence of a thin lubricant layer covering nanostructures, as manifested by the identical contact angles under the general condition and high super-saturation condition.

To characterize the surface wettability, we measured the contact angle on each test surface by the sessile drop method, as shown in Table 1 (Detailed measurement method can be found in the Methods and Supplementary Fig. S2). The measured contact angles under a high supersaturation condition (i.e., flooded condition) were similar with those under the general condition except for SHPo surface, where the measured contact angle under the flooded condition was much smaller than that under the general condition. Although recent studies reported that a high contact angle (>150°) can be maintained under a super-saturation condition on SHPo surfaces having nanoneedles^31^,^33^,^34^, nanopores^35^,^36^ and nanocones^32^,^37^ with the self-removal of droplets by jumping, this behavior was limited to the relatively low super-saturation condition. When a super-saturation level *S* (*S* = *P*_*v*_/*P*_*sat*_ (*T*_*s*_), where *P*_*v*_ is the vapor pressure, *T*_*s*_ is the surface temperature and *P*_*sat*_ (*T*_*s*_) is the saturation pressure at *T*_*s*_) is sufficiently high, the SHPo surface loses its high contact angle as well as its droplet self-removal merit, as the wetting state is transitioned from the non-wetting Cassie state to the flooded Wenzel state. Note that the measured contact angle under flooded condition reflects the actual contact angle during the water harvesting tests, as shown in Figs 3 and 4. In Fig. 3, one can observe numerous tiny droplets (<50 μm in diameter) jumping away from the SHPo surface only in the beginning stage^28^,^38^,^39^,^40^,^41^,^42^. Eventually, a droplet jumping mode disappears, as nanostructures are flooded with the water under the high super-saturation of *S* = 7.5–7.6. In case of oil-infused surfaces as shown in Fig. 4, its low contact angle hysteresis was maintained under flooded condition as shown in Table 1, as a thin lubricant layer protected nanostructures from the water flooding.

### Comparison of fog harvesting and dew harvesting

**Figure 5:**
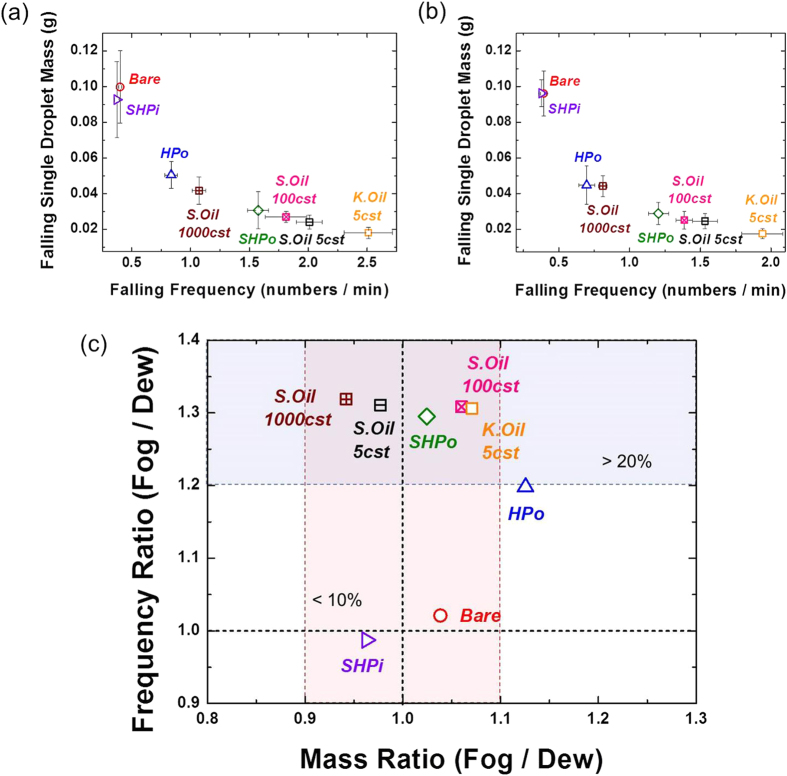
Experimentally measured average mass and frequency of the single falling droplet from each test surface during 90 minutes in (**a**) fog harvesting and (**b**) dew harvesting. As the surface has the better water removal characteristics, the average droplet mass gets smaller. (**c**) Mass ratio and frequency ratio between fog and dew harvesting. While the average mass is similar within about 10% difference under the two test conditions, the falling frequency strongly depends on the harvesting environment for non-wettable surfaces.

In this study, we measured the average mass and frequency of the collected water from each test surface as shown in Fig. 5. The average mass and frequency on each surface were calculated from all the collected droplets during 90 min, at which point they already reached to a near-steady state as shown in Supplementary Fig. S3. In Fig. 5, it can be seen that the falling frequency and average droplet mass varies with the surface wettability and the average droplet mass has the inverse relationship with the falling frequency at both fogging and dewing. Figure 5c compares the frequency and mass of falling droplet under the fogging condition over those under the dewing condition. In this figure, it can be seen that the mass of falling droplet is more or less similar within 10% difference between the two. However, there is a noticeable difference in the falling frequency between the two, particularly for non-wettable surfaces. For example, under the fogging condition, the falling frequency is more than 20–30% higher than that at dewing on oil-infused surfaces and SHPo surface. As will be illustrated in the below, this information can be used to elucidate why the desirable wetting property for fog harvesting can be different from that for dew harvesting.

**Figure 6:**
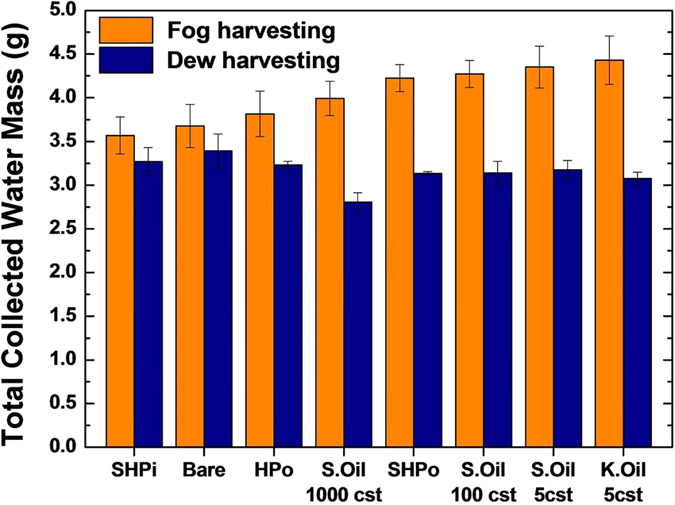
The total amount of collected water on test tube type condensers during 90 minutes in fog harvesting and dew harvesting. In fog harvesting, the water repellent surfaces have the larger amount of the collected water due to the higher water removal efficiency. On the other hand, in dew harvesting, the hydrophilic surface (Bare) shows the best harvesting performance due to its higher nucleation rate.

The total amount of the water collected for duration of 90 minutes is provided in Fig. 6 where the horizontal axis lists the tested surfaces. Figure 6 shows that the better water harvesting performance is associated with the higher falling frequency of droplets on non-wettable surfaces in fog harvesting, while more wettable surfaces such as bare and SHPi are more advantageous for dew harvesting. This result implies that the desirable surface wettability for moisture harvesting is dependent on the way that the moisture in air interacts with the collecting surface.

### Understanding moisture harvesting mechanism

To understand the different performance of each surface in fog harvesting and dew harvesting, it is necessary to look into the detailed mechanism of the moisture harvesting process on each surface. By analogy with the chemical reaction at the surface, one can assume that a moisture harvesting process consists of three stages: the moisture transport onto the surface, its capture on the surface, and the removal of the captured water from the surface, and the water harvesting performance is limited by the least effective one among the three. With a high super-saturation condition used in this study, we can assume that there is enough supply of moisture onto the surface, and then the moisture harvesting will be limited by the least effective one between the moisture capture at the surface and the removal of the captured water from the surface. In fog harvesting, numerous tiny water droplets in air are convected towards the surface and are captured after a direct collision with the surface, while the condensation on the surface is partially responsible for the captured water on the surface as well. Due to a small inertia of the colliding droplet as well as adhesion between the incoming droplet and the surface, most of the incoming droplets will be captured onto the surface irrespectively of the surface wettability. Then, because of a high water capture efficiency by way of a collision, one can expect that the water removal process would be the determining factor to the water harvesting performance in case of fog harvesting. In contrast, in dew harvesting, the water vapor will be captured only via condensation on the surface, which is sensitive to the surface wettability. Then, in dew harvesting, both the water capture rate and removal process could be crucial to the effective moisture harvesting.

### Fog harvesting

**Figure 7:**
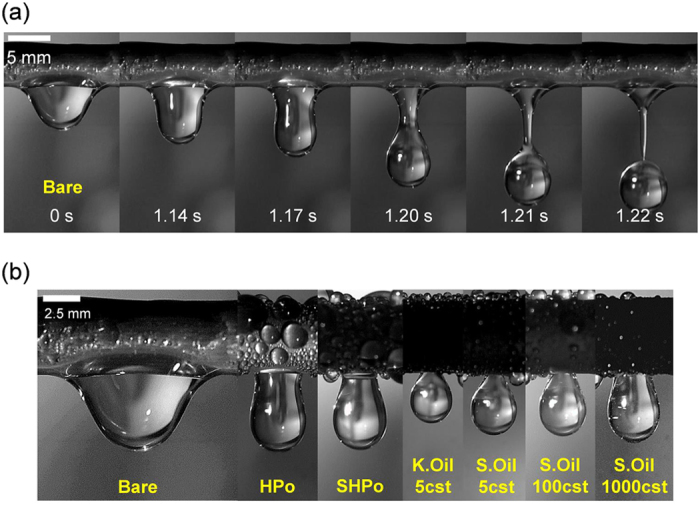
(**a**) Time-lapse images of the drop detachment process on the Bare surface. When a hanging pendant droplet reaches to a critical volume (0 s), it goes through the dynamic detachment process (1.14–1.22 s). This occurs when its weight exceeds the capillary adhesion force. (**b**) Image of the maximum volume of the hanging pendant drop on the Bare, HPo, SHPo, K.Oil 5cst, S.Oil 5cst, S.Oil 100cst, and S.Oil 1000cst surfaces. Each test surface having various wettability exhibits different volume and contact diameter of the hanging pendant drop.

To test the validity of this reasoning, we first quantify the water removal efficiency of each surface based on a capillary adhesion model and correlate it with the fog harvesting performance of each surface. As shown in Fig. 7a, on test surfaces with a tubular geometry, the captured water is transported to the bottom of tube by gravity, forming a hanging drop underneath the tube, and then a water is drained from a hanging drop via dripping. When a hanging drop grows to a critical volume (i.e., corresponding to 0 s in Fig. 7a), its weight exceeds the capillary adhesion force, initiating the dynamic detachment process (i.e., corresponding to 1.14–1.22 s in Fig. 7a). The dynamic detachment process is normally much slower compared with the water transport process to the bottom of the tubular surface, which means that the water detachment from the bottom will govern the overall water removal efficiency of the surface. Still, it happens that the surface with the faster drop transport to the bottom coincides with one with the faster detachment process, although the required condition for each property might be different from each other (See the Supplementary Fig. S4). As the dynamic detachment process is modulated by the capillary adhesion force on each test surface, the surface with different wettability exhibits the different water removal efficiency, as manifested by the different volume and contact diameter of the hanging drop as shown in Fig. 7b.

**Figure 8:**
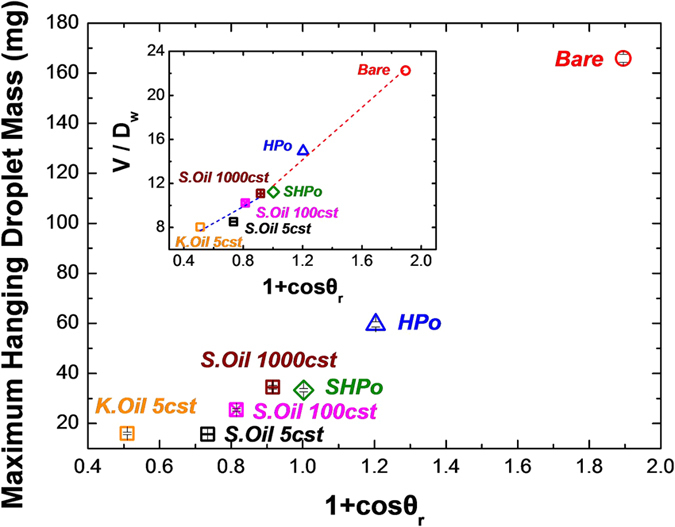
Experimentally measured maximum hanging droplet mass as a function of 1 + *cosθ*_*r*_. Inset: Experimentally measured *V/D*_*w*_ as a function of 1 + *cosθ*_*r*_. The red and blue dashed lines are fitting lines of non-oil-infused surfaces and oil-infused surfaces, respectively. The fitting lines show the linear relation between *V*/*D*_*w*_ and 1 + *cosθ*_*r*_.

In a configuration as shown in Fig. 7, the capillary adhesion force can be modeled as *πD*_*w*_*γ* (1 + *cosθ*_*r*_), where *D*_*w*_ is the contact diameter, *θ*_*r*_ is the receding contact angle, and γ is the surface tension of water^43^. Then, the force balance between the gravitational force (*ρgV: ρ* the water density*, g* the gravitational constant*, V* the drop volume) and this capillary adhesion force yields *ρgV* ~ *πD*_*w*_*γ* (1 + *cosθ*_*r*_), from which we can predict the critical drop size on each surface when the detachment occurs. In case of oil-infused surface, the capillary adhesion force is attributed to the interfacial tension between water and oil, so oil-water interfacial tension *γ*_*ow*_ is used instead of surface tension *γ* in calculating the capillary adhesion force. Figure 8 shows the experimentally measured maximum hanging droplet mass as a function of 1 + *cosθ*_*r*_, while an inset shows the measured *V*/*D*_*w*_ as a function of 1 + *cosθ*_*r*_. In the inset, the red and blue dashed lines are the fitted lines for surfaces without oil-infusion and with oil-infusion, respectively, and they show the linear relation between *V/D*_*w*_ and 1 + *cosθ*_*r*_, as expected from the capillary adhesion model. It needs to be noted that a slope of the red dashed line is approximately 1.6 times larger than that of the blue dashed line, which corresponds to the ratio of surface tension *γ* to oil-to-water interfacial tension *γ*_*ow*_ (i.e., *γ*/*γ*_*ow*_ ~ 1.5). Also, in agreement with the present model, Fig. 8 shows that the oil-infused surfaces with the lowest 1 + *cosθ*_*r*_ exhibit the smallest critical drop size for the drop detachment.

The above analysis indicates that the surface should possess a large receding contact angle for the effective water removal from the surface. Under the general condition, the SHPo surfaces would be the surface of choice for their well-known water repellent properties. However, once the Cassie-to-Wenzel wetting transition occurs under the high super saturation condition, they become less effective for water removal with the drastic decrease of a receding contact angle. Meanwhile, with their robust water repellency, the oil-infused surfaces retain their large receding contact angle under the fogging condition. As a result, the best water harvesting performance is obtained with the oil-infused surfaces prepared with K.Oil or S.Oil 5cst, both of which have the largest receding contact angle. These results support our reasoning that the faster water removal is the key to the efficient fog harvesting.

### Dew harvesting

**Figure 9:**
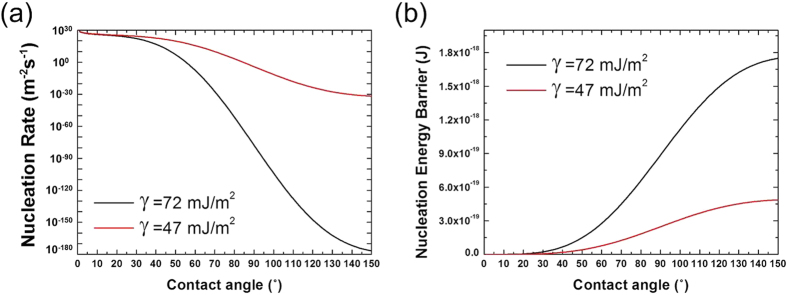
(**a**) Predicted nucleation rates and (**b**) nucleation energy barrier as a function of contact angle and surface energy based on the classical nucleation theory.

Under the dewing condition, wettable surfaces such as Bare and SHPi display the better harvesting performance. In Fig. 5, the comparison of the frequency and mass of the falling droplet between fog and dew harvesting illuminates the underlying cause for this difference. The mass of the falling droplet is directly associated with the water removal efficiency and is independent of the water capture efficiency of each surface. Meanwhile, the frequency of the falling droplet would be a function of both the water removal efficiency and the water capturing rate. In Fig. 5, the similar mass range of falling droplets in fog harvesting and dew harvesting indicates that the water removal characteristics indeed does not vary with the harvesting condition, as the water removal efficiency is a function of the wettability only (represented by a receding contact angle and an interfacial tension). On the other hand, the apparent difference in the falling frequency, particularly for non-wettable surfaces, suggests that the different harvesting performance of each surface at fogging and dewing can be attributed to the different water capture rate under fog harvesting and dew harvesting. At dewing, the water capture rate of each surface is strongly affected by the nucleation rate or nucleation energy barrier of the condensation, which is in itself a function of surface wettability as shown in Fig. 9. In Fig. 9, it can be seen that the more wettable surface has a lower nucleation energy barrier to condensation. For oil-infused surfaces, it has been shown that they may have a higher nucleation rate than other surfaces having similar contact angles ranges because of the low interfacial energy of oil (47 mJ m^−2^)^30^. However, the additional thermal resistance due to the oil layer can offset the benefit of the low interfacial energy of oil (See that the thermal conductivity of liquid water is 0.6 W m^−1^ K^−1^, while that of the Krytox and Silicone oils are only 0.09 and 0.15 W m^−1^ K^−1^, respectively). As a result, the more wettable surfaces (Bare or SHPi) can have the advantage in dew harvesting, as the influence of the water capture efficiency represented by the nucleation rate becomes more important over the influence of the water removal rate. Also, note that the difference of the mass and frequency of the collected droplet between fog harvesting and dew harvesting appear to be rather modest on wettable surfaces of Bare and SHPi (Fig. 5). It means that, on wettable surfaces, the water removal efficiency, which is similar in fog harvesting and dew harvesting, would be the limiting factor to the moisture harvesting performance instead of the water capture efficiency.

Our results suggest that the efficient moisture capture onto the surface and removal from the surface are necessary for the efficient moisture harvesting. At a high water capture rate, as in fog harvesting, the moisture harvesting performance will be limited by the water removal efficiency and increasing the water removal rate by employing non-wettable surfaces such as oil-infused surfaces^21^,^44^,^45^ or facilitating the drop transport by imposing the spatial gradient of Laplace pressure^17^,^19^,^20^ would be the most effective approach to enhance the moisture harvesting performance. On the other hand, for the efficient dew harvesting, it is necessary to have both the efficient water capture rate and removal rate, where the required surface wettability for each contribution is in conflict with each other (more hydrophilic surface for water capture *versus* more hydrophobic surface for water removal). Then, the optimal surface wettability for the dew harvesting will be determined by the subtle balance between the contributions from these two.

In summary, the low wettability is generally desirable for fog harvesting, as it is beneficial to the effective water removal from the surface. But, for dew harvesting, the optimal surface needs to satisfy two conflicting wettability requirements, which might be realized by designing the surfaces with well-controlled heterogeneous wettability or reversible wettability, so that only the advantage from each different wettability can be harnessed.

## Conclusions

In this study, we experimentally investigated the influence of surface wettability on two different modes of moisture harvesting - fog harvesting and dew harvesting. Our results show that the moisture harvesting performance is determined by the combination of the moisture capture at the surface and the removal of the captured water from the surface. In fog harvesting, the moisture capture readily occurs via a direct collision with the surface and thus an efficient water removal, represented by a large receding contact angle, becomes a determining factor to the moisture harvesting performance. Then, the oil-infused surfaces, with their large receding contact angle at a high super-saturation condition, exhibit the best fog harvesting performance. Meanwhile, in dew harvesting, the moisture capture is governed by the nucleation energy barrier to condensation on each surface, which is strongly influenced by the surface wettability. As a result, more wettable surfaces such as Bare or SHPi show the better performance compared to non-wettable surfaces, as the moisture capture efficiency is becoming more important over the water removal efficiency for the water harvesting. Our findings show that the desirable surface wettability for the moisture harvesting are strongly affected by the harvesting condition (whether it is fog harvesting or dew harvesting), as it determines the relative importance of the water capture efficiency and water removal efficiency for the overall harvesting performance. We believe that our results would help design the optimal moisture harvesting surface under the specific harvesting condition by clarifying the influence of surface wettability on the moisture capture and water removal in fog harvesting and dew harvesting.

## Methods

### Water harvesting test setup

The detailed experimental setup used for harvesting water is schematically shown in Supplementary Fig. S1. The test sample was placed horizontally in an acrylic chamber and cool brine from a thermal bath was circulated inside a test tube. In fog harvesting, a hot vapor generated from the boiling water was directly supplied to the test sample and the surface captured the water via a collision with saturated droplets in air as well as via condensation. The air temperature near the test surface was 35.1 ± 1.0 °C, the relative humidity was 90–99%, and the average temperature of brine in the tube was 1.7 ± 0.5 °C. In dew harvesting, there was no supplied vapor stream into the surface and the water was captured only via condensation. In this condition, the air temperature was 40.0 ± 1.0 °C, the relative humidity was ~80%, and the brine temperature inside the tube was 3.4 ± 0.5 °C. The fog and dew harvesting tests were performed in the same super-saturation level of ~7.5 defined as *S* = *P*_*v*_/*P*_*sat*_ (*T*_*s*_), and other parameters were kept as close as possible under the capacity of our experimental setup. For example, all experiments were performed in a temperature and humidity controlled environmental chamber (temperature accuracy of ±0.5 °C and humidity accuracy of ±3.0%, H&C System Korea). The cooling fluid was circulated using a large capacity thermal bath (pump capacity of 22 L/min, pump pressure of 0.47 bar and temperature accuracy of ±0.05 °C at 0 °C, LK Lab Korea). A dripping water droplet from the sample was collected in a clean rectangular vessel placed beneath the sample and the mass and frequency of each falling droplet were measured using an electronic scale and a stopwatch to plot the mass-frequency relationship. Also, we measured the total amount of water collected for 90 min duration, to compare the water harvesting performance of each surface. All images were captured using a high-speed CCD camera.

### Sample fabrication

We prepared several tube type test samples with different wettability from commercially available Cu tubes (99.9% purity, 1.3 mm thickness, 5 mm outer diameter, and 150 mm length). During all chemical fabrication, test tubes were capped by the nipple to prevent any functionalization of the tube inside. Once capped, the sample was cleaned in an ultrasonic bath with acetone for 5 min and rinsed with de-ionized (DI) water at room temperature. Then the tube was dipped into a 2.0 M hydrochloric acid solution for 30 seconds to eliminate the oxide layer on the surface. Once complete, the tube was thoroughly rinsed with DI water and then was dried with clean nitrogen gas. Nanostructured CuO (SHPi) was formed by immersing the cleaned bare Cu tubes inside a hot alkaline solution (95 °C) composed of NaClO_2_, NaOH, Na_3_PO_4_·12 H_2_O, and DI water (3.75: 5:10:100 wt.%) for 10 minutes^26^,^27^. Figure 2 shows the SEM images of such nanostructured CuO structures. To obtain the hydrophobic (HPo) and superhydrophobic (SHPo) surfaces, the bare Cu and nanostructured CuO surfaces were functionalized with TFTS (trichloro(1 H,1 H,2 H,2 H-perfluorooctyl)silane, Sigma) through the vapor deposition process. Oil-infused surfaces were prepared by infusing the Krytox 5cst oil (Dupont) or Silicone oils (XIAMETER, Dow Corning) with different viscosities (5, 100, 1000cst) to SHPo surface using the following procedure. First, the sufficiently large amount of oil was applied to SHPo surface. Then, the excess oil was removed from the surface by using nitrogen gas blowing and then placing the surface vertically for one day to make sure a thin oil layer over the surface.

### Contact angle measurement

Supplementary Fig. S2 shows the schematics of the contact angle measurement procedures in the general condition and flooded condition. As the curvature of Cu tube prevented us from directly measuring the surface wettability using a sessile drop method, we measured a contact angle on the flat Cu plates instead, which were modified using the same method as with Cu tubes. As the measured contact angle can be different depending on the test conditions, particularly for SHPo surfaces under high super-saturation condition due to the transition from the non-wetting Cassie state to the flooded Wenzel state^28^, we measured the contact angles under both the general condition and flooded condition. Contact angles under both conditions were measured by using a sessile drop method, but an additional procedure was performed in flooded condition. First, each sample was placed on a cold plate in a square acrylic chamber and the temperature of the sample was maintained to be ~3 °C by circulating the cooled water from a thermal bath into the block underneath a cold plate. And then hot vapor generated from the boiling water was supplied into the chamber to make the target super-saturation condition of 7.5, which was used in the actual water harvesting test. Then, after gently blowing away condensed droplets near a target water drop with a nitrogen gas, we measured the flooded contact angle. As shown in Table 1, while the contact angles of Bare and HPo surfaces were only slightly smaller under flooded condition, there was a noticeable drop in the measured contact angles, particularly the receding contact angle, on the SHPo surface due to the flooding of nanostructures by a high amount of moisture in air. Meanwhile, the oil-infused surfaces maintained their original contact angles as well as the low contact angle hysteresis under flooded condition.

## Additional Information

**How to cite this article**: Seo, D. *et al.* The effects of surface wettability on the fog and dew moisture harvesting performance on tubular surfaces. *Sci. Rep.* **6**, 24276; doi: 10.1038/srep24276 (2016).

## Supplementary Material

Supplementary Information

## Figures and Tables

**Table 1:** Contact angles of bare Cu (Bare), hydrophobic (HPo), superhydrophilic (SHPi), superhydrophobic (SHPo), 5cst krytox oil-infused superhydrophobic (K.Oil 5cst), 5cst silicone oil-infused superhydrophobic (S.Oil 5cst), 100cst silicone oil-infused superhydrophobic (S.Oil 100cst), and 1000cst silicone oil-infused superhydrophobic (S.Oil 1000cst) surfaces in general and flooding conditions.

Samples	Advancing (°)		Static (°)		Receding (°)	
	General	Flooded	General	Flooded	General	Flooded
Bare	101.5 ± 3.8	87.3 ± 2.4	89.6 ± 3.5	79.5 ± 2.3	30.9 ± 4.3	26.5 ± 2.3
HPo	131.9 ± 2.2	129.6 ± 1.7	116.1 ± 1.3	111.1 ± 1.5	81.2 ± 5.4	78.3 ± 2.3
SHPi	<10	<10	<10	<10	0	0
SHPo	162.7 ± 1.2	132.7 ± 4.2	161.7 ± 4.0	121.6 ± 3.3	161.1 ± 1.1	89.8 ± 2.4
K.Oil 5cst	123.8 ± 2.1	121.4 ± 2.6	122.0 ± 3.4	120.4 ± 1.2	121.6 ± 2.1	119.4 ± 1.5
S.Oil 5cst	109.6 ± 2.4	106.3 ± 3.7	108.3 ± 2.8	106.1 ± 2.5	107.3 ± 2.5	105.4 ± 4.2
S.Oil 100cst	105.3 ± 1.8	102.5 ± 2.0	104.2 ± 2.6	101.0 ± 3.6	103.9 ± 2.7	100.7 ± 2.1
S.Oil 1000cst	104.3 ± 2.3	102.6 ± 3.1	101.6 ± 4.3	100.2 ± 3.9	100.7 ± 2.7	94.8 ± 2.6
