# Supplementary material for: The effects of surface wettability on the fog and dew moisture harvesting performance on tubular surfaces
Source: Sci Rep. 2016 Apr 11;6:24276. doi: 10.1038/srep24276 (PMC4827126; doi:10.1038/srep24276)
Supplement: Supplementary Information [file srep24276-s1.doc]

**Supporting Information**

**The effects of surface wettability on the fog and dew moisture harvesting performance on tubular surfaces**

# Donghyun Seo^1^, Junghun Lee^1^, Choongyeop Lee^1,*^, and Youngsuk Nam^1,*^

^1^Department of Mechanical Engineering, Kyung Hee University, Yongin 446-701, Korea

*cylee@khu.ac.kr, *ysnam1@khu.ac.kr


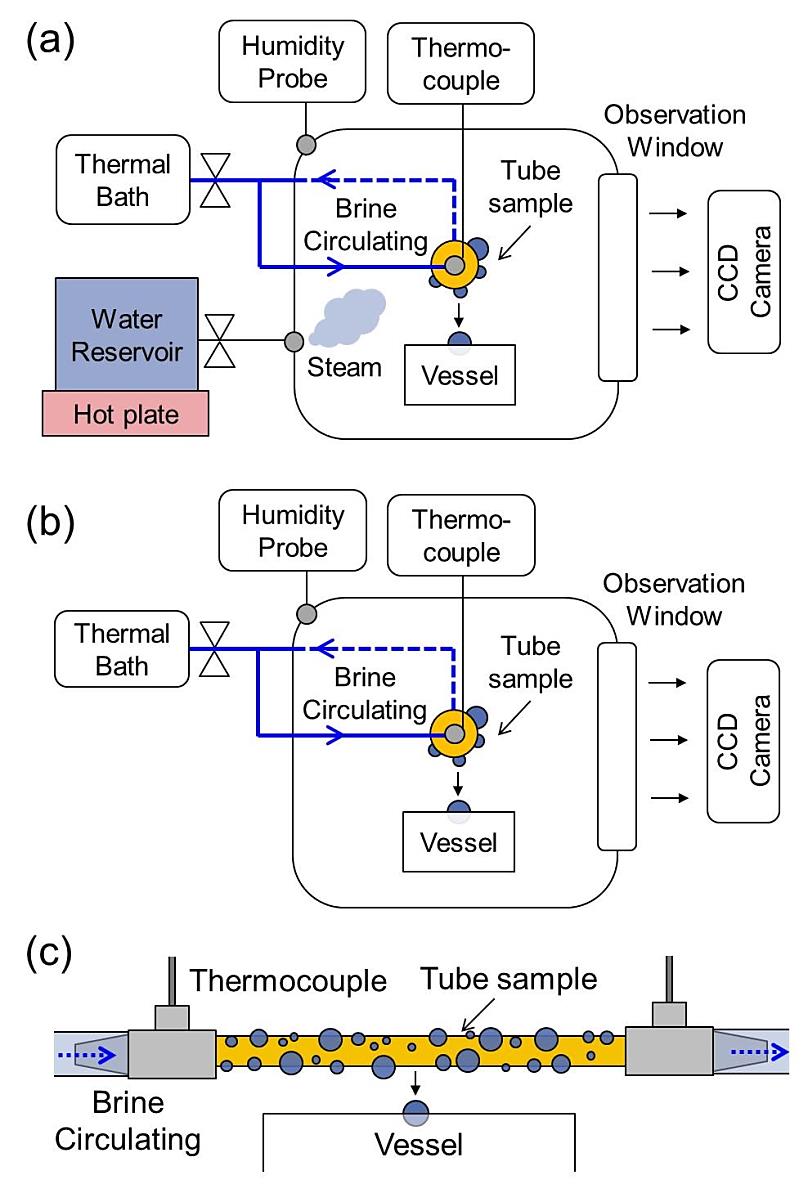


**Figure S1**. Schematics of the water harvesting setup (a) under the steam condition and (b) dew condition. (a) In the steam, hot vapor generated from the boiling water is supplied directly to the test sample, while (b) in the dew, the direct contact between hot vapor and the test surface is avoided. The temperature and humidity are controlled by the environmental chamber while maintaining the similar super-saturation conditions under both conditions. (c) Schematic showing the installed test tube inside the chamber.


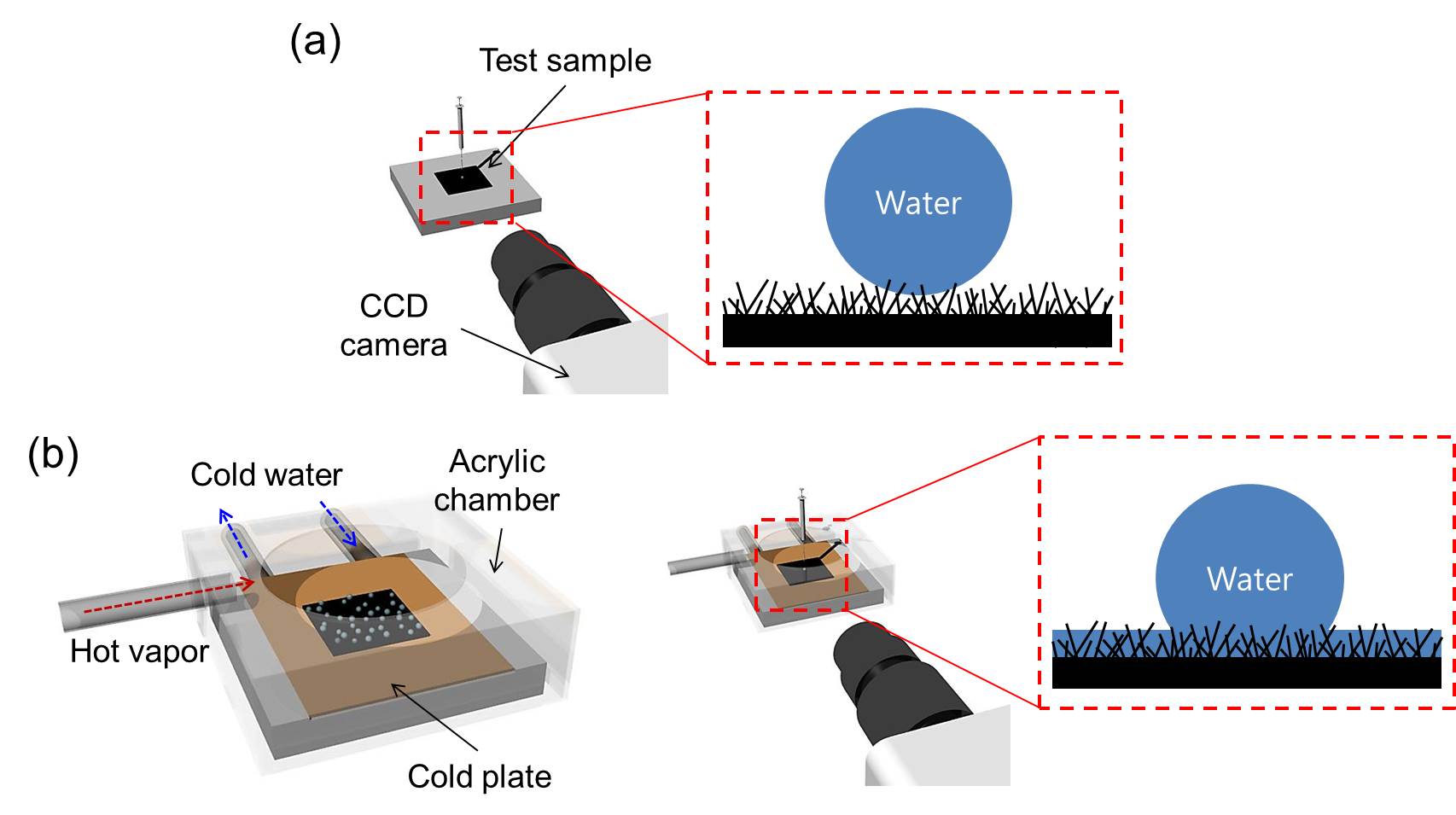


**Figure S2**. Schematics of contact angle measurement procedure under (a) general and (b) flooded conditions. (a) Under the general condition, a droplet is placed on a test sample using a micro syringe and is imaged by a CCD camera for contact angle measurement. (b) Under the flooded condition, the test sample is placed on a cold plate within an acrylic chamber, while a hot vapor generated from a boiling water is supplied into the chamber to maintain the same super-saturation level (*S* = 7.5) as the actual moisture harvesting experiment. After the sample is exposed to the moisture for the duration of 30 min, a droplet is placed on a test sample using a micro syringe and is imaged by a CCD camera for contact angle measurement.


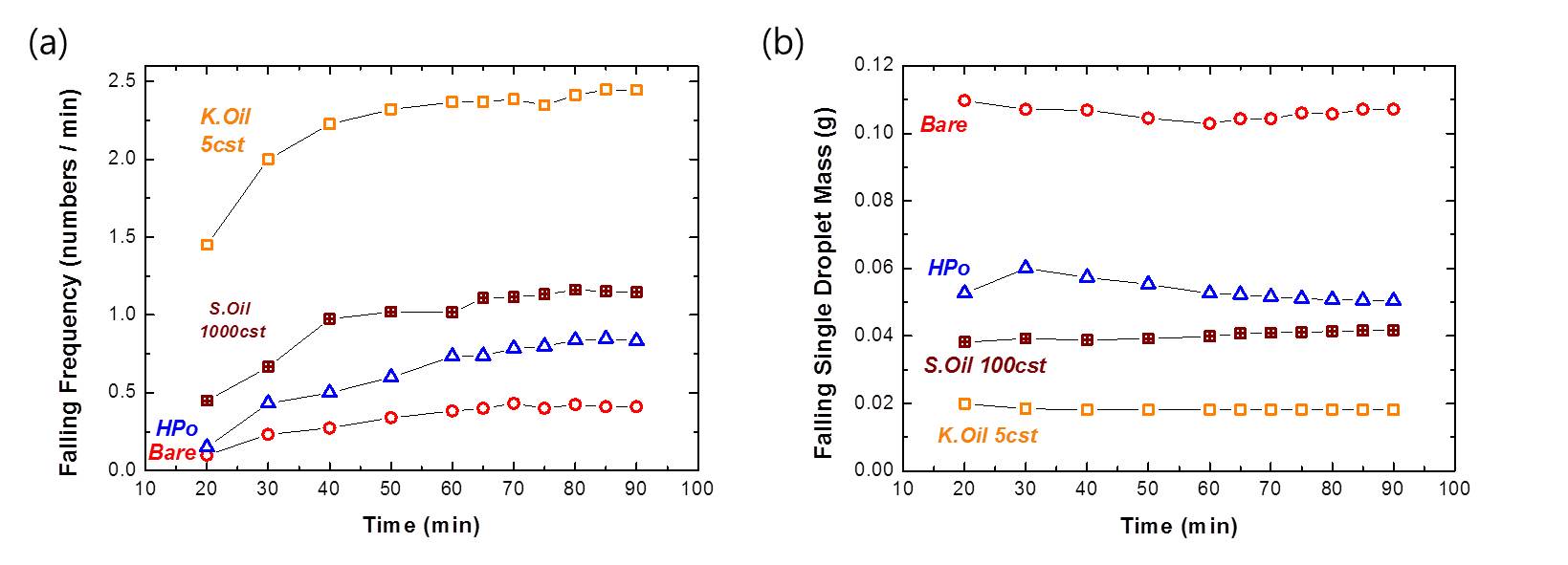


**Figure S3**. (a) Averaged falling frequency and (b) single droplet mass of all the collected droplets as a function of time on Bare, HPo, S.Oil 1000cst, and K.Oil 5cst surfaces under the fog condition. Each point in the figure is the average value of all the collected droplets from the begging (0 s) up to the designated time in *x*-axis. In the beginning of the experiments (< ~10 min), there are no falling droplets, as the size of captured droplets on the surfaces is not large enough to fall by gravity. After ~10 min, the frequency is increasing over time, as more and more droplets begin to fall by gravity. After 60 min, the averaged falling frequency reaches a near-steady value, as the number of sampled droplets is statistically large enough. (b) The average mass of the collected droplets remains relatively constant with time, as the mass of falling droplets is solely determined by the surface wettability.


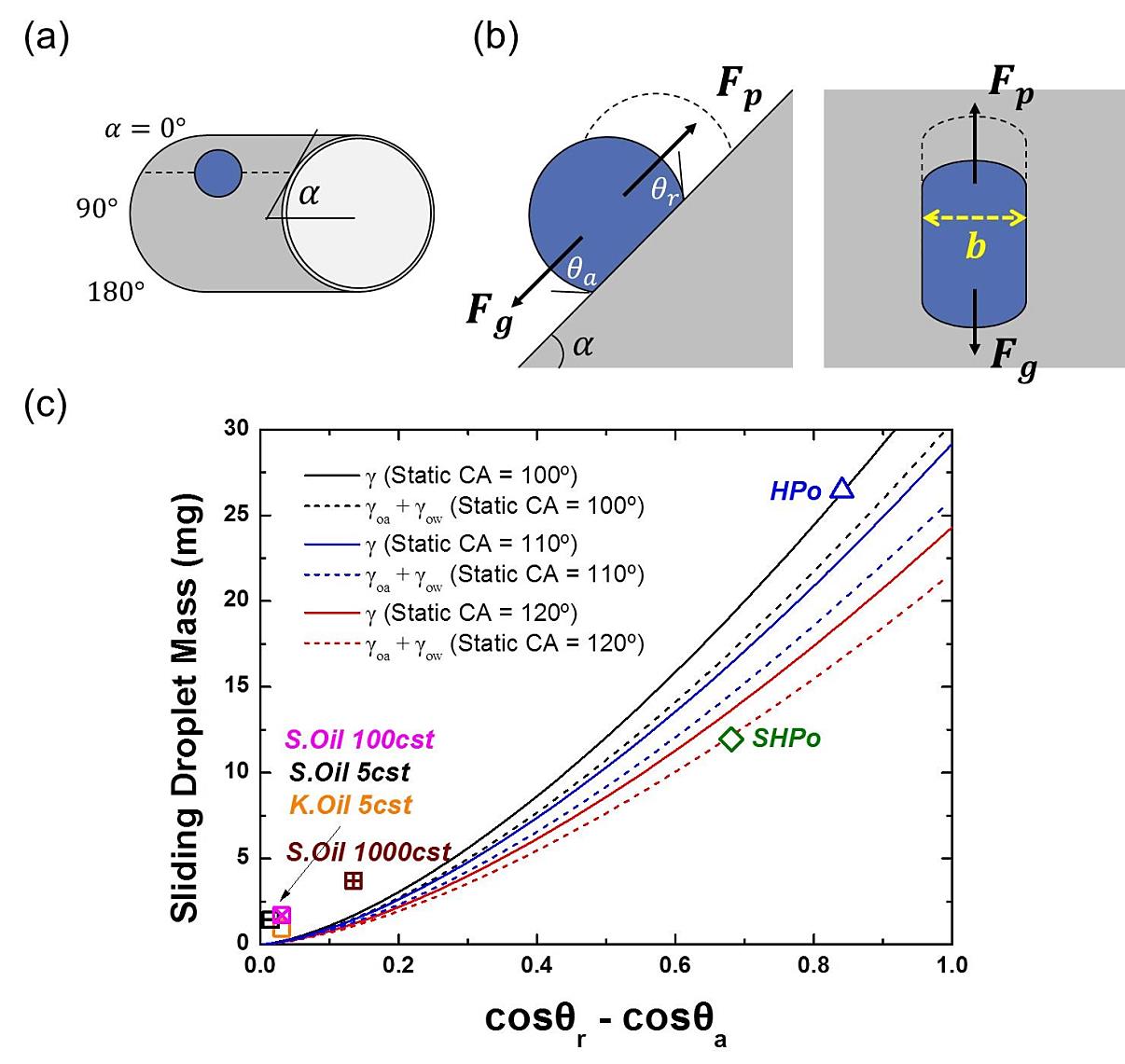


**Figure S4**. Schematics of (a) the sliding droplet on the surface and (b) the force balances acting on the droplet. When the gravitational force (*F_g_*) of the droplet overcomes the pinning force (*F_p_*), the droplet begins to slide down on the surface. (c) The sliding droplet mass as a function of contact angle hysteresis at a tilt angle of 90º. In case of the oil-infused surfaces, the sum of oil-air and oil-water interfacial tensions replaces water-air surface tension because of oil-layer cloaking a water droplet. The low contact angle hysteresis leads to the smaller sliding droplet mass.


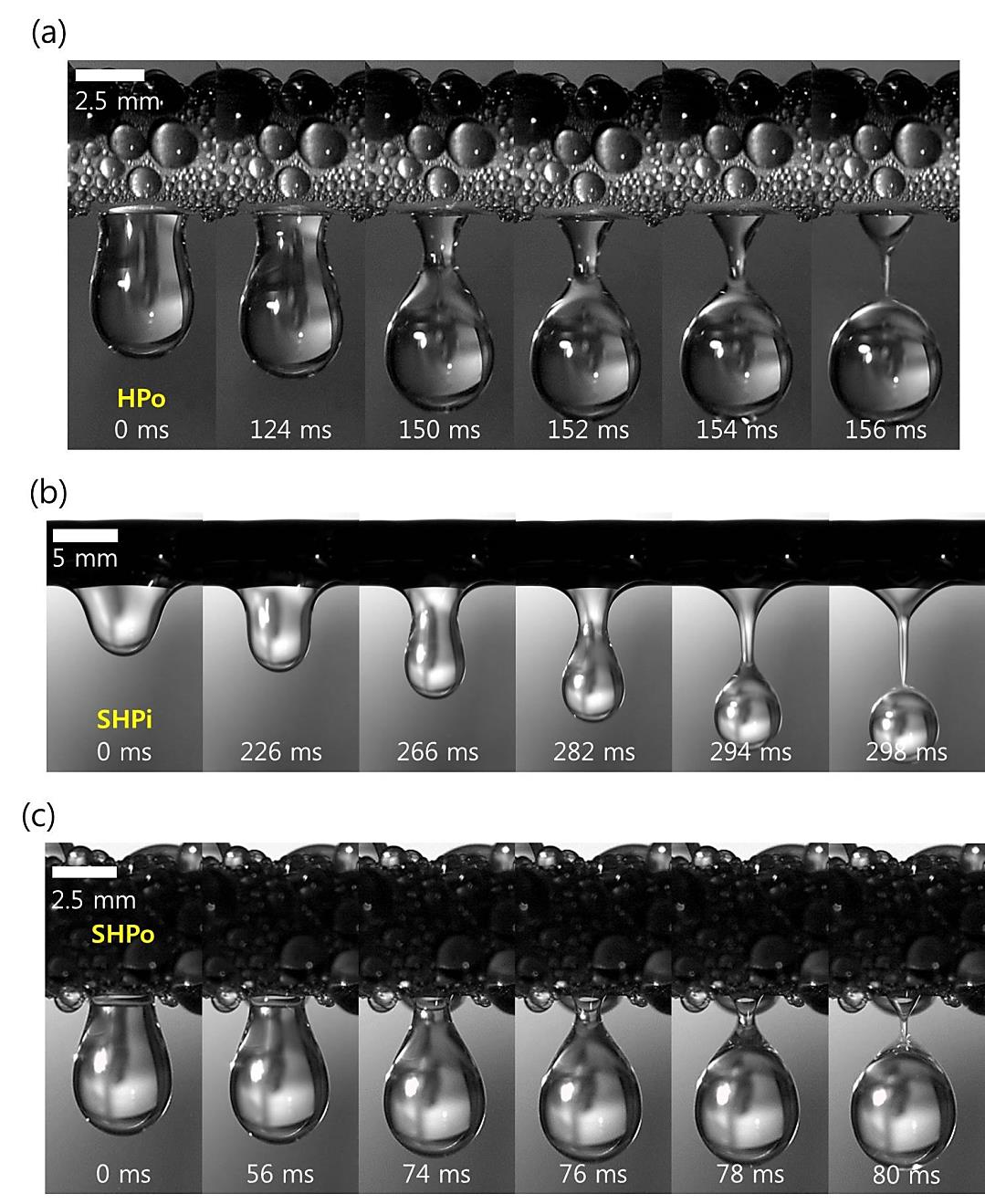


**Figure S5**. Time-lapse images of the dripping process on the (a) HPo, (b) SHPi, and (c) SHPo surfaces.


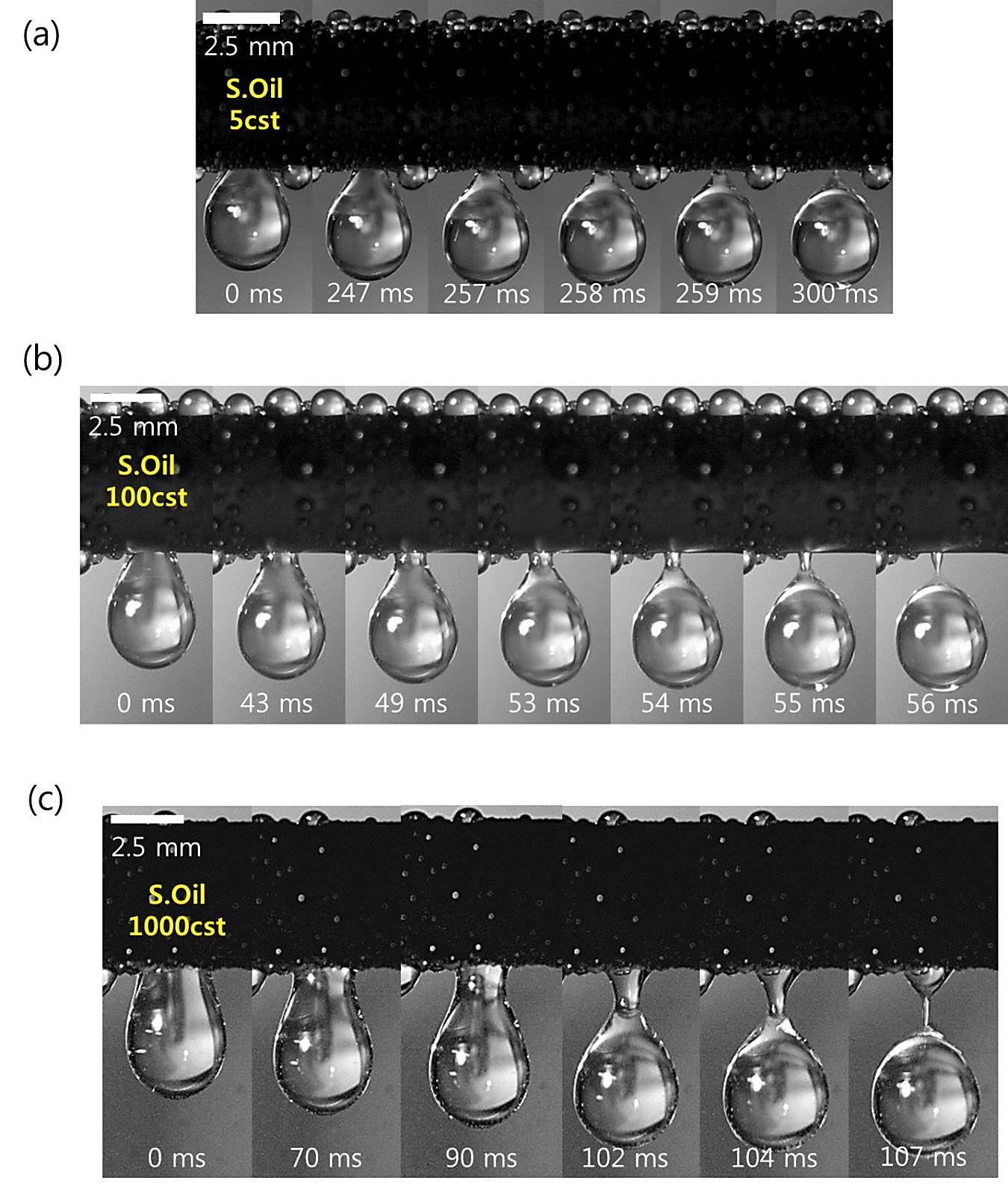


**Figure S6**. Time-lapse images of the dripping process on the (a) S.Oil 5cst, (b) S.Oil 100cst, and (c) S.Oil 1000cst surfaces.
